# Smoking, e-cigarettes and the effect on respiratory symptoms among a population sample of youth: Retrospective cohort study

**DOI:** 10.18332/tid/156839

**Published:** 2023-01-21

**Authors:** Michael Chaiton, Martha Pienkowski, Iman Musani, Susan J. Bondy, Joanna E. Cohen, Jolene Dubray, Thomas Eissenberg, Pamela Kaufman, Matthew Stanbrook, Robert Schwartz

**Affiliations:** 1 Dalla Lana School of Public Health, University of Toronto, Toronto, Canada; 2 Centre for Addiction and Mental Health, Toronto, Canada; 3 Institute for Global Tobacco Control, Department of Health, Behavior and Society, Johns Hopkins Bloomberg School of Public Health, Baltimore, United States; 4 Department of Psychology, Virginia Commonwealth University, Richmond, United States; 5 Division of Respirology, Department of Medicine, University of Toronto, Toronto, Canada

**Keywords:** smoking, respiratory health, youth, e-cigarettes

## Abstract

**INTRODUCTION:**

E-cigarettes have been steadily increasing in popularity, both as cessation methods for smoking and for recreational and social reasons. This increase in vaping may pose cardiovascular and respiratory risks. We aimed to assess respiratory symptoms in youth users of e-cigarettes and cigarettes.

**METHODS:**

A retrospective survey design was utilized to assess Canadian youth aged 16–25 years. Participants were recruited from the Ontario Tobacco Research Unit Youth and Young Adult Research Registration Panel November 2020 to March 2021. A total of 3082 subjects completed the baseline survey. Of these, 2660 individuals who did not have asthma were included in the analysis. The exposure of interest was pack-equivalent years, a novel measure of vaping exposure equivalent conceptually to cigarette pack years incorporating number of puffs per day, number of days vaped per month, and number of years vaped. Respiratory symptoms were measured using the five-item Canadian Lung Health Test. Poisson regression analyses were performed while adjusting for demographic confounders, stratified by smoking status. A non-stratified model tested the interaction of status and vaping dose and the effect of vaping device used was assessed among ever vapers. Analyses controlled for demographic characteristics, use of cannabis and alcohol, and survey date.

**RESULTS:**

Each additional puff year increased the rate ratio (RR) of respiratory symptoms by a factor of 11.36 (95% CI: 4.61–28.00; p<0.001) for never smokers, but among current daily smokers higher pack-equivalent years were not associated with more respiratory symptoms (RR=0.83; 95% CI: 0.23–3.11). Among current vapers, those using pod-style devices were more likely to have more respiratory symptoms (RR=1.25; 95% CI: 1.08–1.45) after adjusting for dose.

**CONCLUSIONS:**

Vaping is associated with an increased risk of reporting respiratory symptoms among never smoking youth and non-daily ever cigarette smokers. Use of e-cigarettes among non-smokers should be discouraged.

## INTRODUCTION

Cigarette smoking has long been known to be associated with poor respiratory health outcomes, including lung cancer, chronic obstructive pulmonary disease (COPD), and severe asthma^1^. In the past several decades, the prevalence of cigarette smoking has decreased drastically across North America and Europe. Even so, it remains prevalent with an estimated 14.8% of Canadians smoking cigarettes in 2019, which is slightly higher than rates of 14.1% in the United Kingdom and 14.0% in the United States^2-4^. While the use of cigarettes has become less common, electronic cigarettes (e-cigarettes) have quickly gained global popularity as both smoking cessation aids as well as recreational nicotine delivery devices^5^. This rise in use has been especially prominent amongst youth, with several studies observing large increases in the prevalence of youth e-cigarette use in both the US and Canada within the past decade, with 15% of youth in Canada aged 15–19 years reporting past 30-day use of e-cigarettes in 2019, compared to only 6% in 2017^6,7^. Rates of e-cigarette use are also increasing globally although prevalence rates are relatively low compared to North America and Europe^8^.

E-cigarettes have been found to be associated with some health-related harms^9,10^. Literature surrounding the health impacts of e-cigarette use is still evolving, but existing research has identified both short- and long-term respiratory harms due to e-cigarette use^11^. For older smokers looking to use e-cigarettes to quit smoking, it is likely that the magnitude of these potential harms is less of a concern compared to use of e-cigarettes among youth who are more at risk of long-term health effects due to e-cigarette use.

Amongst the potential health effects of vaping among youth, respiratory symptoms are most likely to become evident early on. Symptoms of chronic bronchitis and asthma in young people have been associated with lung function decline amongst older people^12^. NASEM (2018) identifies several ways in which the nicotine in e-cigarette aerosol might damage the respiratory system as well as how small particulate matter and flavorings might independently impair lung function. For example, upon being heated, saccharides that are used in making sweet e-cigarette juice flavors breakdown, producing furans and aldehydes that can irritate the respiratory tract. Harms to the respiratory system from e-cigarette use may be more likely when initiation begins at a young age.

Research surrounding the health impacts of the dual use of cigarettes and e-cigarettes remains limited. Results from existing studies have been mixed, with some finding that dual use leads to mitigated health-related harms due to the overall decrease in the use of cigarettes^13^. Other research has found that the potential mitigation of health-related harms as a result of switching from cigarettes to e-cigarettes are negated in those who continue to smoke cigarettes alongside using e-cigarettes. This may be observed as no difference in health-related harms between dual users and single product users or increased harms among dual users. Notably, most of these studies focus on the health-related impacts of either smoking or vaping, but not both^14-18^. Finally, much research remains inconclusive surrounding the harms associated with the dual use of cigarettes and e-cigarettes, as well as with regard to how these impacts differ between individuals who are solely users of either cigarettes or e-cigarettes^12^.

The current analysis sought to use survey data to understand better the association between e-cigarette use and self-reported respiratory symptoms among youth. In doing so, we seek to add to a growing body of research regarding the extent of harms related to the use of e-cigarettes. Additionally, the study aims to assess the interaction associated with the dual use of cigarettes and e-cigarettes on respiratory symptoms. Based on current research on both vaping and smoking cigarettes, we hypothesized that individuals who smoked cigarettes regularly and those who vaped regularly would report more respiratory symptoms compared to non-smokers and non-vapers. We further hypothesized that dual use of cigarettes and e-cigarettes would be associated with more respiratory symptoms compared to vaping alone, but lower compared to cigarette smoking alone, due to satiation of cravings that would decrease cigarettes smoked daily. However, this assumption of decreased cigarette intake may be incorrect, as some studies reported lower levels of smoking in dual users, while others reported no notable change in number of cigarettes smoked, though fewer respiratory symptoms were still observed^18^. Further, we examined the effect of vaping device type after controlling for vaping exposure.

## METHODS

### Data source

Participants were recruited from the Ontario Tobacco Research Unit Youth and Young Adult Research Registration Panel November 2020 to March 2021 (Pienkowski, 2021). Panel recruitment was completed via social media advertisements from August 2020 to February 2021. Panel participants completed a recruitment survey and provided information for future contact. From the panel, adolescents and young adults aged 16–25 years living in Canada were eligible for the survey. Panel participants were invited to participate in this study sequentially using quota sampling to balance the survey respondents for vaping status [never, ever (at least one puff), past 30 days], smoking status (never, ever, past 30 days) and age (16–18 and 19–25 years). Those who consented to participate in this study then completed a self-administered online questionnaire. This study used a cross-sectional design. The University of Toronto Research Ethics Board provided ethical approval.

### Measures

The primary exposure variable was pack-equivalent years. This novel measure combines self-reported assessments of number of times the respondent takes a puff on a vape per day they vape (‘When you vape, how many puffs do you take?’), number of days vaped in the past month (‘On how many days, of the past 30 days, did you vape?’), and number of years since vaping started, calculated as current age subtracting reported age when the participant first vaped (‘How old were you when you first tried vaping?’). The pack-equivalent years measure is equivalent to the pack-years calculation for cigarettes. The number of reported puffs per day on days vaped (divided by 10, the standard number of puffs in a cigarette) was multiplied by the number of days vaped per month (divided by 30 to provide an average daily use). This value was then divided by 20 to convert to packs (20 cigarettes per pack) and then multiplied by the number of years of vaping report.

The outcome variable was the self-reported occurrence of adverse respiratory symptoms. Data on five respiratory symptoms were collected, with survey respondents identifying any symptoms they experienced in the past four months (yes/no). The five symptoms were coughing regularly, coughing up phlegm regularly, feeling out of breath from even simple chores, wheezing when exerting oneself (e.g. through exercise or going up the stairs), and getting many colds (specifically those that take longer to recover). The five questions were combined to form an overall respiratory symptoms variable, the Canadian Lung Health Test. This measure was originally validated for use in screening for COPD but comprises a set of questions routinely used by clinicians to evaluate patients for respiratory disorders^19^. The maximum number of respiratory symptoms that could be selected by a respondent was five and the minimum number of symptoms was zero.

Cigarette status was self-reported and categorized into (never smoked a cigarette, smoked a cigarette but not a current daily smoker, and daily smoker). Participants also reported number of cigarettes smoked per day, and whether or not they were past month users of alcohol and cannabis. Participants were asked what vaping device they had last used, and how long they had been vaping at the time of survey completion. Data on demographic characteristics were also collected (sex, age, education level, parental status, marital status, province of residence, and race). Participants also reported whether or not they had received a diagnosis of asthma.

### Statistical analysis

This analysis assessed the effect of e-cigarette use and frequency on the rate ratios (RRs) of respiratory symptoms, assessed via a Poisson regression model. The interaction between e-cigarette puffs per day and number of cigarettes smoked per day on respiratory symptoms was also assessed. All other covariates in the model were chosen based on past research. Covariates were included in the analysis if they were deemed to be conceptually relevant as confounders. Covariates were respondent age, sex, race, education level, race, province and history of substance-use other than cigarettes or e-cigarettes.

An additional Poisson regression analysis was conducted which assessed effects of self-reported vaping device type, vaping flavor, and number of years since starting vaping on respiratory symptoms among current e-cigarette users. Analyses were performed using Stata/IC 16.1.

## RESULTS

Participation rate was 60%. Younger females, those with higher education, and those who had never used e-cigarettes or cigarettes were more likely to participate. There were 3082 youth who completed the baseline survey including 396 individuals reporting a diagnosis of asthma. Descriptive statistics were calculated for all variables included in the Poisson regression model and outlined in Supplementary file Table 1a. The average age of participants included in this analysis was 19.6 years (SD=2.7), ranging 16–25 years. The majority of respondents were White (n=2270; 73.7%) and 80.6% were female (n=2456); 396 individuals reporting a diagnosis of asthma.

Half of respondents reported either never vaping or not currently vaping at all (n=1554; 50.4%), 10.5% reported less than monthly vaping (n=323), 5.4% reported vaping monthly (n=165), 5.8% vaped weekly (n=178), and 28.0% reported vaping daily (n=862). The average number of cigarettes smoked per day among ever-smokers in the sample was 4.4 (SD=7·2), while the daily average among exclusive current daily smokers was 9.8 (SD=11.8) and the daily average among current daily dual users was 5.7 (SD=5.9) cigarettes per day. Never smokers who vaped had lower numbers of puffs per day, number of days vaped per month, and number of years vaped than ever smokers and daily smokers (Table 1).

**Table 1:** Mean number of puffs per day vaped, number of days per month vaped, number of years vaped, by smoking status (never, ever, and daily) among ever vapers aged 16–25 years, Canada, 2020–2021 (N=2150)

	*Never smokers (n=840)*		*Ever smokers (except daily) (n=1118)*		*Daily smokers (n=192)*	
	*Mean*	*SD*	*Mean*	*SD*	*Mean*	*SD*
Number of puffs per day	3.2	0.2	5.9	0.2	7.8	0.7
Number of days vaped	6.4	0.4	12.6	0.4	13.6	1
Number of years vaped	1.7	0.1	2.4	0.1	2.2	0.2

Table 2 outlines the percentage distribution of respiratory symptoms by smoking and vaping status. Half of respondents reported zero respiratory symptoms (51.5%). Of never users, non-daily and daily e-cigarette users, the highest proportion of respondents reported zero symptoms (65.8%, 56.8%, and 34.2%, respectively), compared to the highest proportion of daily smokers who reported experiencing one respiratory symptom (23.2%), and the highest proportion of dual users who reported experiencing two respiratory symptoms (25.0%). While for three of the five symptoms the majority of participants did not report experiencing the symptom, regardless of smoking or vaping status, the majority of daily smokers, regardless of vaping status, did report coughing regularly in the last four months (65.6% of daily smokers, non-vapers; 57.1% of daily smokers, daily vapers), and the majority of daily smokers, non-vapers reported coughing up phlegm regularly (52.2%). Additionally, daily smokers, daily vapers, and daily dual users were all more likely to report experiencing any of the five symptoms compared to non-daily or never users of either cigarettes or e-cigarettes. Daily smokers were also more likely to report experiencing any symptoms compared to daily vapers. Dual users had lower rates of reporting of all symptoms compared to daily smoking alone, especially when looking at wheezing (35.8% of dual users, compared to 46.1% of daily smokers) (p<0.05 for cold frequency and duration, p<0.001 for all other respiratory symptoms and total number of symptoms).

**Table 2:** Descriptive statistics of percentage of participants with respiratory symptoms by smoking and vaping status, age 16–25 years, Canada, 2020–2021 (N=3082)

	*Total n (%)*	*Never use of both smoking and vaping n (%)*	*Non-daily ever use of either smoking or vaping n (%)*	*Daily vaping, non-daily/never smoking n (%)*	*Daily smoking, non-daily/never vaping n (%)*	*Daily vaping, daily smoking n (%)*	*p*
**Total**	3082	825	1303	820	92	42	
**Number of respiratory symptoms** (N=2973)							<0.001
0	1531 (51.5)	534 (65.8)	712 (56.8)	266 (34.2)	12 (13.3)	7 (17.5)	
1	683 (23.0)	168 (20.7)	277 (22.1)	209 (26.9)	21 (23.3)	8 (20.0)	
2	425 (14.3)	76 (9.4)	166 (13.2)	156 (20.1)	17 (18.9)	10 (25.0)	
3	186 (6.3)	21 (2.6)	66 (5.3)	77 (9.9)	17 (18.9)	5 (12.5)	
4	111 (3.7)	11 (1.4)	23 (1.8)	53 (6.8)	16 (17.8)	8 (20.0)	
5	37 (1.2)	1 (0.1)	10 (0.8)	17 (2.2)	7 (7.8)	2 (5.0)	
**Over the past 4 months, did you cough regularly?** (N=2999)							<0.001
Yes	686 (22.9)	81 (9.9)	207 (16.4)	315 (40.1)	59 (65.6)	24 (57.1)	
**Over the past 4 months, did you cough up phlegm regularly?** (N=2996)							<0.001
Yes	509 (17.0)	67 (8.2)	170 (13.4)	206 (26.3)	47 (52.2)	19 (45.2)	
**Over the past 4 months, did even simple chores make you short of breath?** (N=2992)							<0.001
Yes	629 (21.0)	94 (11.5)	231 (18.3)	242 (30.9)	41 (45.6)	21 (51.2)	
**Over the past 4 months, did you wheeze when you exerted yourself?** (N=2995)							<0.001
Yes	673 (22.5)	135 (16.5)	254 (20.1)	223 (28.4)	43 (47.8)	18 (43.9)	
**Over the past 4 months, did you get many colds and do your colds usually last longer than your friends’ colds?** (N=2999)							<0.05
Yes	247 (8.2)	61 (7.5)	97 (7.6)	70 (8.9)	15 (16.7)	4 (9.8)	

Each additional puff year increased the rate ratio of respiratory symptoms by a factor of 11.36 (95% CI: 4.61–28.00; p<0.001) for never smokers and ever smokers (RR=2.79; 95% CI: 1.69–4.61), but among current daily smokers higher pack-equivalent years were not associated with more respiratory symptoms (RR=0.83; 95% CI: 0.23–3.11) (Table 3). Test of interactions were statistically significant (p<0.001).

**Table 3:** Poisson regression summary table of rate ratios (RR) for effects of vaping and smoking frequencies on respiratory symptoms stratified by smoking status^a^, Canada, 2020–2021

	*Non-smokers only*	*Ever smokers only*	*Daily smokers only*	*All participants*	*Among current vapers (device type analysis)*
	*RR (95% CI)*	*RR (95% CI)*	*RR (95% CI)*	*RR (95% CI)*	*RR (95% CI)*
**Pack-equivalent years^b^**	11.36 (4.61–28.00)***	2.79 (1.69–4.61)***	0.84 (0.23–3.11)***	2.29 (1.41–371)***	2.20 (1.34–3.61)**
**Smoking status**					
Ever smoker (Ref.)					
Daily smoker				1.47 (1.24–1.73)***	1.53 (1.29-1.82)
Never smoker				0.68 (0.61–0.76)***	0.75 (0.66–0.85)***
**Interactions**					
Daily smoker X pack-equivalent years				0.41 (0.11–1.55)	0.37 (0.66–0.85)
Never smoker X pack-equivalent years				4.26 (1.62–11.20)**	3.10 (1.11–8.72)
**Device type**					
Cig-a-like device					1.03 (0.86–1.24)
Pod type device					1.25 (1.08–1.45)**
N	1445	1059	156	2660	1795

a All analysis show exponentiated coefficients after adjustment for respondent age, sex, education level, marital status, parental status, registration date, other daily drug use, province, and race; those who reported a diagnosis of asthma are excluded. Full tables with covariates available in (Supplementary file Table 1).

b Pack-equivalent years is calculated as the number of reported puffs per day on days vaped (divided by 10, the standard number of puffs in a cigarette), multiplied by the number of days vaped per month (divided by 30 to provide an average daily use), then divided by 20 to convert to packs (20 cigarettes per pack) and finally multiplied by the number of years of vaping self-report (current age – age at when first started vaping).

* p<0.05.

** p<0.01.

*** p<0.001.

**Figure 1:**
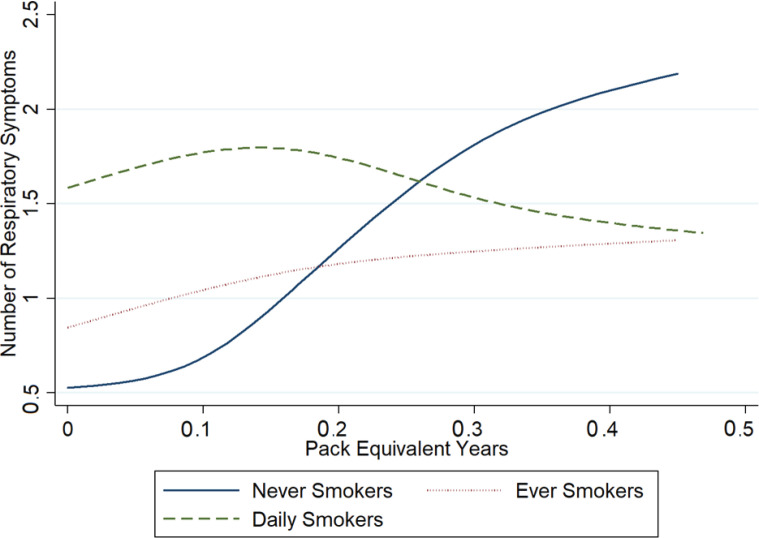
Modelled number of respiratory symptoms by e-cigarette pack-equivalent years by smoking status among Canadian youth (aged 16–25 years) (N=2660)

Figure 1 depicts the modelled relationship between pack-equivalent years and number of respiratory symptoms, by cigarette smoking status. Never smokers with no vaping history have fewer respiratory symptoms than those who have smoked with average number of symptoms of respiratory symptoms approaches the level seen in daily smokers after 0.5 puff year. Daily smokers do not demonstrate increasing symptoms with greater pack-equivalent years. Among current vapers, those using pod-style devices were more likely to have more respiratory symptoms (RR=1.25; 95% CI: 1.08–1.45) after adjusting for dose (Table 3).

## DISCUSSION

Our analysis found that use of e-cigarettes was associated with increased rate of respiratory symptoms, and that the greater the frequency of vaping the higher the number of symptoms. Unsurprisingly, higher amounts of cigarette smoking were also strongly associated with more respiratory symptoms. Cumulative exposure to vaping based on vaping frequency and length of time vaping was associated with increased numbers of respiratory symptoms that can, with sufficient dose, approach the short-term respiratory harms associated with smoking cigarettes.

This population of youth and young adults reflects a lighter smoking sample than would be found among all adults. Exclusive daily smokers reported smoking an average of 9.8 cigarettes per day, while dual users reported smoking an average of 5.7 cigarettes per day, which is notably lower than previous research that showed an average daily cigarette consumption among Canadian daily smokers of 13.7 in 2017^20^. Despite this level of smoking, elevated respiratory symptoms were higher in smokers than non-smokers indicating the dangerousness of cigarettes.

Our study is consistent with previous evidence. A recent review of the literature found that on average studies showed a significant association of e-cigarette use with asthma and COPD, controlling for cigarette smoking and other covariates. For asthma (n=15 studies), the pooled adjusted odds ratio (AOR) was 1.39 (95% CI: 1.28–1.51); for COPD (n=9 studies) the AOR was 1.49 (95% CI: 1.36–1.65)^21,22^. Any use of vaping in short- or long-term was associated with asthma in youth aged 15–16 years^23^.

With regard to dual use, however, we observed that those individuals who vaped daily and smoked cigarettes daily did not demonstrate additional risk from vaping. That is, dual users reported notably high levels of respiratory symptoms compared to non-users of both cigarettes and e-cigarettes, though an increase in e-cigarette puffs resulted in a marginal decrease in rate of respiratory symptoms. These results are congruent with existing studies that reported health benefits of dual use compared to exclusive cigarette smoking^12,13,15,17^. However, unlike this current study, a study of over 45000 secondary school students in Hong Kong found that e-cigarette users had significantly higher odds of having respiratory symptoms than non-e-cigarette users regardless of smoking status^21^. Amongst never smoking e-cigarette users, the adjusted odds ratio for respiratory symptoms was 2.06 (95% CI: 1.24–3.42). This interaction between e-cigarette and cigarette use requires further study as many individuals engage in dual use of cigarettes and e-cigarettes^12,13^. Motivations for dual use vary, with research suggesting that individuals who initiate the use of e-cigarettes with the goal of smoking cessation are more likely to engage in dual use than to engage in e-cigarette use alone^12^. This may be the case because smokers who use e-cigarettes as smoking cessation aids may not fully transition from smoking to vaping, despite often beginning with the intention to quit smoking entirely^12^. However, due to the high baseline of respiratory symptoms among cigarette smokers, it is difficult to ascertain the true benefit of dual use without longitudinally monitoring changes in smoking behaviors of exclusive smokers that become dual users, to determine the mechanism behind this decrease in symptoms.

This study also builds on previous results via the inclusion of a vaping-only group, as well as via the inclusion of youth participants, which allows for more specified results and interpretations. Though the specification of age may limit generalizability to the overall population, it also allows for increased accuracy and reliability in constructing prevention and treatment plans for youth. Additional studies that monitor exact number of cigarettes smoked and e-cigarette puffs among single users versus dual users are needed to confirm this.

Those who vaped pod like devices reported higher levels of symptoms compared to ‘mod’-type devices after controlling for puff year. This finding is consistent with previous results wherein adolescents reported experiencing worse respiratory symptoms when using specific vape brands and products, particularly JUUL, a pod device with a nicotine-salt liquid. As was expected, subjects with a longer history of vaping experienced more respiratory symptoms than newer vapers, as they have likely inhaled a larger number of total puffs.

### Limitations

While this analysis provided insight into the association between the dual use of vaping and smoking on respiratory health symptoms, there are limitations that should be noted. First, there are substantial limitations to the generalizability of this survey due to the purposive online sampling. The high participation of females suggests that the results are more robust for young females. While the anonymous, online nature of the survey may encourage honesty, there is also a risk of recall bias, as participants may incorrectly recall their use behaviors or respiratory symptoms, either unintentionally or intentionally, especially if they are advocates for smoking or vaping. There are fewer numbers of individuals who are daily smokers compared to never or ever smokers, and the level of number of cigarettes per daily among this group of relatively young daily smokers is light compared to older or more representative samples. Additional studies that include physical assessments of subjects’ respiratory health and biomarkers of smoking/vaping are necessary to achieve more accurate and reliable results.

The retrospective nature of these data also presents some limitations with regard to the conclusions that can be drawn from our analysis. Because survey respondents were asked about their use of cigarettes and e-cigarettes simultaneously and retrospectively, it is unclear how patterns of use changed over the life course and how these reported symptoms may or may not lead to medical diagnosis of disease in the long-term. Finally, this study introduces a new measure of exposure to vaping, pack-equivalent years, which is based conceptually on cigarette pack equivalent measures that have been used in respiratory and health effects study of cigarettes for decades. There is little consensus on the best measure to assess exposure to vaping and so consequently we have attempted to include as much information available on exposure, dose, and frequency as we have available. Nevertheless, this measure may be more or less biased than other measures for exposure to vaping.

## CONCLUSIONS

This study documented a potential correlation between vaping and an increase in respiratory symptoms. A secondary finding was an interaction between dual use of cigarettes and e-cigarettes and a decreased risk of respiratory symptoms compared to cigarette smoking alone, which exhibited a higher baseline of symptoms. Further longitudinal research using physical health assessments and biomarkers is necessary to explore this association further.

## Data Availability

The data supporting this research are available from the authors on reasonable request.
