# Supplementary material for: Smoking, e-cigarettes and the effect on respiratory symptoms among a population sample of youth: Retrospective cohort study
Source: Tob Induc Dis. 2023 Jan 21;21:08. doi: 10.18332/tid/156839 (PMC9865633; doi:10.18332/tid/156839)
Supplement: Supplementary file 1 [file TID-21-08-s1.pdf]

Supplemental Table 1a. Demographics for all variables included in the multivariable linear regression model, Canada, 2020-21, age 16-25. N=3082.

	Total	Never-use of both smoking and vaping	Non-daily ever-use of either smoking or vaping	Daily vaping, non-daily/never smoking	Daily smoking, non-daily/never vaping	Daily vaping, daily smoking
	N=3,082	N=825	N=1,303	N=820	N=92	N=42
**Age**	19.6 (2.7)	18.8 (2.6)	20.1 (2.8)	19.6 (2.5)	21.6 (2.6)	19.6 (2.5)
**16-19**	1,689 (54.8%)	561 (68.0%)	634 (48.7%)	448 (54.6%)	22 (23.9%)	24 (57.1%)
**20-25**	1,393 (45.2%)	264 (32.0%)	669 (51.3%)	372 (45.4%)	70 (76.1%)	18 (42.9%)
**Sex**						
**Male**	593 (19.4%)	116 (14.2%)	219 (17.0%)	227 (28.0%)	17 (18.9%)	14 (33.3%)
**Female**	2,456 (80.6%)	701 (85.8%)	1,071 (83.0%)	583 (72.0%)	73 (81.1%)	28 (66.7%)
**Race**						
**White**	2,270 (73.7%)	494 (59.9%)	983 (75.4%)	682 (83.2%)	78 (84.8%)	33 (78.6%)
**Black**	62 (2.0%)	22 (2.7%)	32 (2.5%)	8 (1.0%)	0 (0.0%)	0 (0.0%)
**Chinese**	201 (6.5%)	109 (13.2%)	72 (5.5%)	20 (2.4%)	0 (0.0%)	0 (0.0%)
**Filipino**	43 (1.4%)	18 (2.2%)	15 (1.2%)	10 (1.2%)	0 (0.0%)	0 (0.0%)
**Indigenous**	63 (2.0%)	5 (0.6%)	26 (2.0%)	22 (2.7%)	6 (6.5%)	4 (9.5%)
**Japanese**	4 (0.1%)	0 (0.0%)	3 (0.2%)	1 (0.1%)	0 (0.0%)	0 (0.0%)
**Korean**	19 (0.6%)	7 (0.8%)	7 (0.5%)	4 (0.5%)	1 (1.1%)	0 (0.0%)
**Latin-Central-South American**	55 (1.8%)	12 (1.5%)	29 (2.2%)	11 (1.3%)	2 (2.2%)	1 (2.4%)
**Southeast Asian**	38 (1.2%)	21 (2.5%)	12 (0.9%)	5 (0.6%)	0 (0.0%)	0 (0.0%)

**South Asian**	182 (5.9%)	99 (12.0%)	65 (5.0%)	16 (2.0%)	2 (2.2%)	0 (0.0%)
**West Asian or Arab**	56 (1.8%)	14 (1.7%)	30 (2.3%)	9 (1.1%)	1 (1.1%)	2 (4.8%)
**Other**	89 (2.9%)	24 (2.9%)	29 (2.2%)	32 (3.9%)	2 (2.2%)	2 (4.8%)
**Province**						
**Ontario**	1,531 (49.7%)	478 (57.9%)	664 (51.0%)	322 (39.3%)	49 (53.3%)	18 (42.9%)
**Alberta**	496 (16.1%)	104 (12.6%)	203 (15.6%)	168 (20.5%)	15 (16.3%)	6 (14.3%)
**British Columbia**	483 (15.7%)	111 (13.5%)	210 (16.1%)	144 (17.6%)	10 (10.9%)	8 (19.0%)
**Manitoba**	116 (3.8%)	25 (3.0%)	52 (4.0%)	32 (3.9%)	6 (6.5%)	1 (2.4%)
**New Brunswick**	53 (1.7%)	15 (1.8%)	14 (1.1%)	22 (2.7%)	2 (2.2%)	0 (0.0%)
**Newfoundland and Labrador**	40 (1.3%)	6 (0.7%)	16 (1.2%)	17 (2.1%)	0 (0.0%)	1 (2.4%)
**Nova Scotia**	102 (3.3%)	27 (3.3%)	47 (3.6%)	24 (2.9%)	2 (2.2%)	2 (4.8%)
**Prince Edward Island**	16 (0.5%)	2 (0.2%)	5 (0.4%)	9 (1.1%)	0 (0.0%)	0 (0.0%)
**Quebec**	129 (4.2%)	28 (3.4%)	59 (4.5%)	34 (4.1%)	3 (3.3%)	5 (11.9%)
**Saskatchewan**	113 (3.7%)	27 (3.3%)	33 (2.5%)	47 (5.7%)	5 (5.4%)	1 (2.4%)
**Northwest Territories**	2 (0.1%)	1 (0.1%)	0 (0.0%)	1 (0.1%)	0 (0.0%)	0 (0.0%)
**Yukon**	1 (0.0%)	1 (0.1%)	0 (0.0%)	0 (0.0%)	0 (0.0%)	0 (0.0%)
**Highest education level**						
**Some elementary or high school**	764 (24.8%)	286 (34.7%)	261 (20.0%)	185 (22.6%)	21 (22.8%)	11 (26.2%)
**Completed High school**	1,489 (48.3%)	356 (43.2%)	604 (46.4%)	469 (57.2%)	40 (43.5%)	20 (47.6%)
**College diploma**	268 (8.7%)	33 (4.0%)	133 (10.2%)	79 (9.6%)	18 (19.6%)	5 (11.9%)
**University or post graduate degree**	561 (18.2%)	150 (18.2%)	305 (23.4%)	87 (10.6%)	13 (14.1%)	6 (14.3%)
**Marital status**						

Single	2,577 (83.6%)	768 (93.1%)	1,058 (81.2%)	658 (80.2%)	63 (68.5%)	30 (71.4%)
Married or living with a partner	501 (16.3%)	55 (6.7%)	244 (18.7%)	162 (19.8%)	28 (30.4%)	12 (28.6%)
Divorced/Separated/ Widowed	4 (0.1%)	2 (0.2%)	1 (0.1%)	0 (0.0%)	1 (1.1%)	0 (0.0%)
**Parental status**						
Yes	60 (1.9%)	4 (0.5%)	28 (2.1%)	20 (2.4%)	8 (8.7%)	0 (0.0%)
No	3,022 (98.1%)	821 (99.5%)	1,275 (97.9%)	800 (97.6%)	84 (91.3%)	42 (100.0%)
**Vaping device type (last used)**						
Modifiable/tubular device	331 (15.9%)		180 (15.6%)	122 (15.2%)	23 (25.8%)	6 (14.3%)
Pod system	1,426 (68.3%)		753 (65.1%)	599 (74.9%)	47 (52.8%)	27 (64.3%)
Other[1]	331 (15.9%)		224 (19.4%)	79 (9.9%)	19 (21.3%)	9 (21.4%)
**E-liquid flavour (last used)**						
Fruit/Candy/Dessert/Food	1,351 (64.0%)		737 (62.5%)	531 (66.5%)	54 (60.7%)	29 (69.0%)
Beverage	35 (1.7%)		23 (1.9%)	9 (1.1%)	1 (1.1%)	2 (4.8%)
Mint/Menthol	387 (18.3%)		175 (14.8%)	192 (24.0%)	13 (14.6%)	7 (16.7%)
Tobacco	59 (2.8%)		23 (1.9%)	25 (3.1%)	8 (9.0%)	3 (7.1%)
Other	82 (3.9%)		42 (3.6%)	34 (4.3%)	5 (5.6%)	1 (2.4%)
I don't know	196 (9.3%)		180 (15.3%)	8 (1.0%)	8 (9.0%)	0 (0.0%)
**How often do you currently vape?**						

**Daily or almost daily**	862 (28.0%)	0 (0.0%)	0 (0.0%)	820 (100.0%)	0 (0.0%)	42 (100.0%)
**Less than daily, but at least once a week**	178 (5.8%)	0 (0.0%)	154 (11.8%)	0 (0.0%)	24 (26.1%)	0 (0.0%)
**Less than weekly, but at least once a month**	165 (5.4%)	0 (0.0%)	150 (11.5%)	0 (0.0%)	15 (16.3%)	0 (0.0%)
**Less than monthly**	323 (10.5%)	0 (0.0%)	302 (23.2%)	0 (0.0%)	21 (22.8%)	0 (0.0%)
**Not at all**	622 (20.2%)	0 (0.0%)	593 (45.5%)	0 (0.0%)	29 (31.5%)	0 (0.0%)
**I have never vaped**	932 (30.2%)	825 (100.0%)	104 (8.0%)	0 (0.0%)	3 (3.3%)	0 (0.0%)
**Cigarettes smoked daily**	4.4 (7.2)		1.9 (3.0)	3.2 (4.7)	9.8 (11.8)	5.7 (5.9)
**Daily alcohol/cannabis use**	878 (28.5%)	30 (3.6%)	348 (26.7%)	420 (51.2%)	51 (55.4%)	29 (69.0%)
**Asthma diagnosis**	396 (12.8%)	107 (13.0%)	154 (11.8%)	106 (12.9%)	19 (20.7%)	10 (23.8%)

[1]Other devices: disposable or rechargeable cigarette-like vaping devices, pen-like devices, and other.

Supplement Table 1. Poisson Regression predicting respiratory symptoms among youth.					
Outcome: Respiratory Symptoms	Among Never Smokers	Among Ever Smokers	Among Daily Smokers	Full population	Device Type
Pack Equivalent Years	11.36***	2.79***	0.84	2.29***	2.20**
	[4.61,28.00]	[1.69,4.61]	[0.23,3.11]	[1.41,3.71]	[1.34,3.61]
Age					
16-19	0.95*	0.97	1.04	0.98	0.98
20-25	[0.90,1.00]	[0.94,1.01]	[0.98,1.11]	[0.95,1.00]	[0.95,1.01]
Sex					
Male	1.42***	1.38***	1.09	1.35***	1.33***
Female	[1.15,1.74]	[1.19,1.62]	[0.79,1.51]	[1.20,1.51]	[1.18,1.50]
Education					
High school or more	1.03	0.84***	0.82*	0.91**	0.89**
	[0.91,1.16]	[0.76,0.92]	[0.68,1.00]	[0.85,0.97]	[0.82,0.96]
Marital Status					
Married or living with a partner	0.91	1.00	0.78	0.95	0.96
	[0.71,1.17]	[0.85,1.19]	[0.58,1.05]	[0.84,1.07]	[0.84,1.09]
Divorced/Separated	2.40	1.31	1.00	1.63	1.17
	[0.74,7.81]	[0.41,4.21]	[1.00,1.00]	[0.71,3.70]	[0.37,3.74]
Parent	1.01	0.93	1.21	0.95	0.84
	[0.52,1.98]	[0.63,1.37]	[0.58,2.52]	[0.71,1.29]	[0.61,1.15]
Survey Date	1.00	1.00	1.00	1.00	1.00
	[1.00,1.00]	[1.00,1.00]	[1.00,1.00]	[1.00,1.00]	[1.00,1.00]
Past Month Cannabis Use	1.99***	1.78***	1.27	1.78***	1.71***
	[1.66,2.39]	[1.57,2.02]	[0.96,1.68]	[1.62,1.96]	[1.55,1.89]
Past Month Alcohol Use	1.29	1.20	0.99	1.19*	1.22*
	[0.92,1.82]	[0.98,1.46]	[0.68,1.44]	[1.02,1.38]	[1.04,1.42]
Province (ref: Ontario)					
Alberta	0.99	1.08	1.34	1.10	1.10
	[0.80,1.22]	[0.91,1.28]	[0.92,1.95]	[0.97,1.24]	[0.96,1.26]
British Columbia	0.90	1.03	1.06	1.00	0.99
	[0.73,1.12]	[0.86,1.22]	[0.69,1.62]	[0.88,1.14]	[0.86,1.14]

Manitoba	1.40	0.66*	0.90	0.94	0.92
	[0.99,1.98]	[0.45,0.97]	[0.38,2.10]	[0.74,1.20]	[0.70,1.21]
New Brunswick	2.15***	1.22	2.24*	1.64***	1.38*
	[1.43,3.24]	[0.82,1.80]	[1.18,4.26]	[1.27,2.12]	[1.02,1.88]
Newfoundland and Labrador	1.19	1.01	1.00	1.14	1.13
	[0.69,2.05]	[0.63,1.60]	[1.00,1.00]	[0.81,1.62]	[0.78,1.63]
Nova Scotia	1.06	1.08	1.40	1.09	0.99
	[0.70,1.61]	[0.74,1.58]	[0.82,2.40]	[0.85,1.40]	[0.75,1.31]
Prince Edward Island	0.00	1.66	0.00	1.21	1.47
	[0.00,.]	[0.97,2.84]	[0.00,.]	[0.71,2.06]	[0.86,2.50]
Quebec	1.03	0.86	2.02**	1.08	1.04
	[0.70,1.51]	[0.63,1.18]	[1.25,3.25]	[0.87,1.33]	[0.82,1.31]
Saskatchewan	1.10	0.92	2.06**	1.16	1.08
	[0.77,1.57]	[0.66,1.29]	[1.23,3.47]	[0.93,1.44]	[0.85,1.38]
Northwest Territories	1.00	1.00	1.00	1.00	1.00
	[1.00,1.00]	[1.00,1.00]	[1.00,1.00]	[1.00,1.00]	[1.00,1.00]
Yukon	1.89	1.00	1.00	1.88	1.00
	[0.26,13.46]	[1.00,1.00]	[1.00,1.00]	[0.26,13.40]	[1.00,1.00]
Race (ref: White)					
Black	1.09	0.86	3.04	1.06	1.08
	[0.73,1.62]	[0.49,1.53]	[0.83,11.12]	[0.77,1.44]	[0.74,1.56]
Chinese	0.78	0.70	1.00	0.73**	0.64*
	[0.59,1.03]	[0.43,1.11]	[1.00,1.00]	[0.57,0.92]	[0.45,0.90]
Filipino	1.04	1.40	1.00	1.13	1.00
	[0.60,1.81]	[0.72,2.72]	[1.00,1.00]	[0.74,1.72]	[0.59,1.70]
Indigenous	1.53	0.91	0.89	1.00	0.98
	[0.86,2.72]	[0.61,1.36]	[0.48,1.65]	[0.75,1.33]	[0.73,1.31]
Japanese	5.42***	0.00	1.00	2.03	2.26
	[2.21,13.31]	[0.00,.]	[1.00,1.00]	[0.84,4.90]	[0.93,5.46]
Korean	0.34	1.19	1.09	0.74	1.01
	[0.08,1.36]	[0.49,2.88]	[0.25,4.72]	[0.39,1.43]	[0.52,1.95]

Latin-Central-South American	1.32	0.95	0.87	1.11	1.12
	[0.83,2.11]	[0.59,1.54]	[0.31,2.45]	[0.81,1.52]	[0.80,1.56]
Southeast Asian	0.97	0.58	1.00	0.80	0.67
	[0.56,1.69]	[0.26,1.30]	[1.00,1.00]	[0.51,1.26]	[0.33,1.34]
South Asian	1.16	1.27	1.41	1.18	1.31*
	[0.91,1.49]	[0.89,1.80]	[0.61,3.25]	[0.97,1.44]	[1.00,1.70]
West Asian or Arab	1.23	0.72	0.50	0.96	0.83
	[0.80,1.89]	[0.37,1.39]	[0.17,1.45]	[0.69,1.35]	[0.56,1.22]
Other	1.00	0.86	1.03	0.94	0.93
	[0.65,1.53]	[0.59,1.24]	[0.55,1.96]	[0.73,1.22]	[0.71,1.22]
Number of cigarettes per day				1.02***	1.01**
				[1.01,1.02]	[1.01,1.02]
Smoking status (ref: Ever Smoker)					
Daily Smoker				1.47***	1.53***
				[1.24,1.73]	[1.29,1.82]
Never Smoker				0.68***	0.75***
				[0.61,0.76]	[0.66,0.85]
Interactions					
Daily smoker X pack equivalent years				0.41	0.37
				[0.11,1.55]	[0.10,1.45]
Never smoker X pack equivalent years				4.26**	3.10*
				[1.62,11.20]	[1.11,8.72]
Device Type (re: mod)					
Cig-a-like device					1.03
					[0.86,1.24]
Pod type device					1.25**
					[1.08,1.45]
N	1445	1059	156	2660	1795

Exponentiated coefficients; 95% confidence intervals in brackets

* $p < 0.05$, ** $p < 0.01$, *** $p < 0.001$

© 2023 Chaiton M. et al.
